# A case of periostomy intestinal metaplasia without adenomatous or dysplastic changes in an ulcerative colitis patient

**DOI:** 10.1002/ccr3.2678

**Published:** 2020-02-13

**Authors:** Nneka S. Udechukwu, Maria A. Selim, Matilda W. Nicholas

**Affiliations:** ^1^ Department of Dermatology Duke University Medical Center Durham North Carolina; ^2^ Department of Pathology Duke University Medical Center Durham North Carolina

**Keywords:** benign cellular transformation, complications, inflammatory bowel disease, metaplasia, ostomy

## Abstract

We strive to educate medical providers of the possibility of cellular transformation occurring as a parastomal complication and to emphasize the importance of close monitoring, as there is a risk, although low, of subsequent malignant transformation.

## Graphical abstract

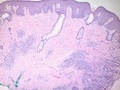

## 1 INTRODUCTION

A rare case of a 52‐year‐old man with multiple comorbidities including a history of ulcerative colitis and subsequent total proctocolectomy with end ileostomy was presented for the evaluation of persistent papules around his stoma. Biopsy confirmed squamous epithelium with areas transitioning to enteric mucosa consistent with intestinal metaplasia around his ostomy site.

Intestinal metaplasia is an uncommon benign cellular transformation that can occur in patients with an ostomy.^1^, ^2^, ^3^, ^4^ The etiology remains elusive, but chronic irritation and trauma have been suggested as possible risk factors.^5^ We present an uncommon case of intestinal metaplasia without adenomatous or dysplastic changes arising along the periphery of an ostomy site in a patient with ulcerative colitis.

## 2 REPORT OF A CASE

**Figure 1:**
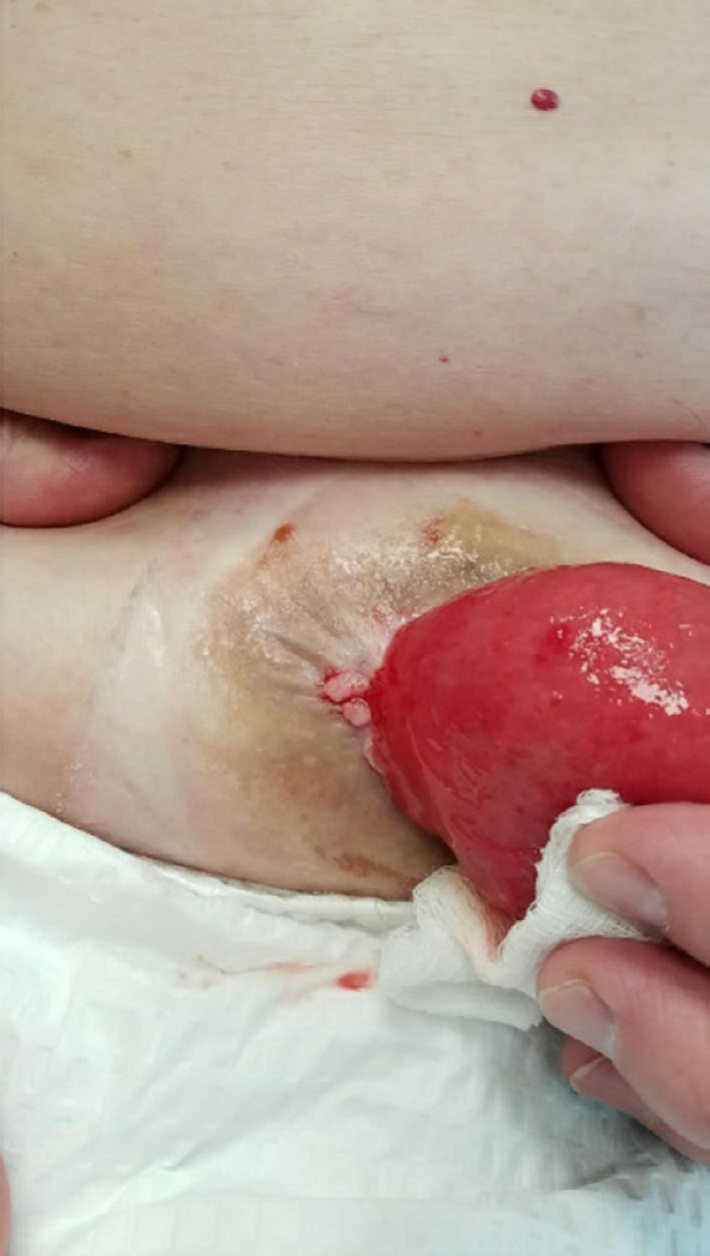
Hyperkeratotic polypoid papules on the lateral aspect of the ileostomy site in right lateral abdomen

**Figure 2:**
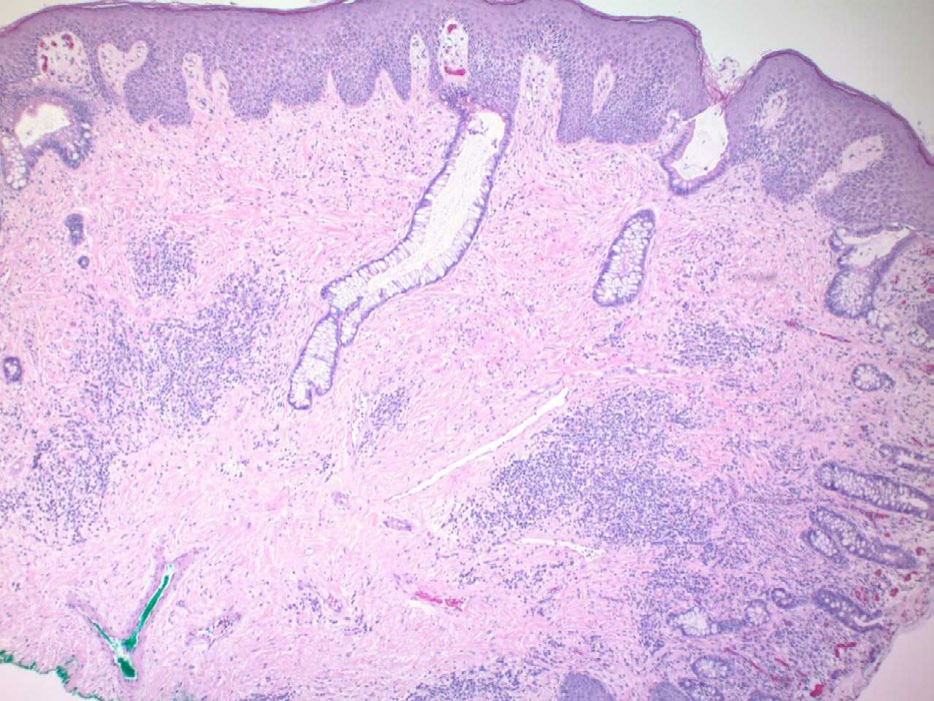
Hematoxylin‐eosin staining of biopsy specimen showing colonic glands merging with the squamous epithelium of the skin (magnification 40×)

**Figure 3:**
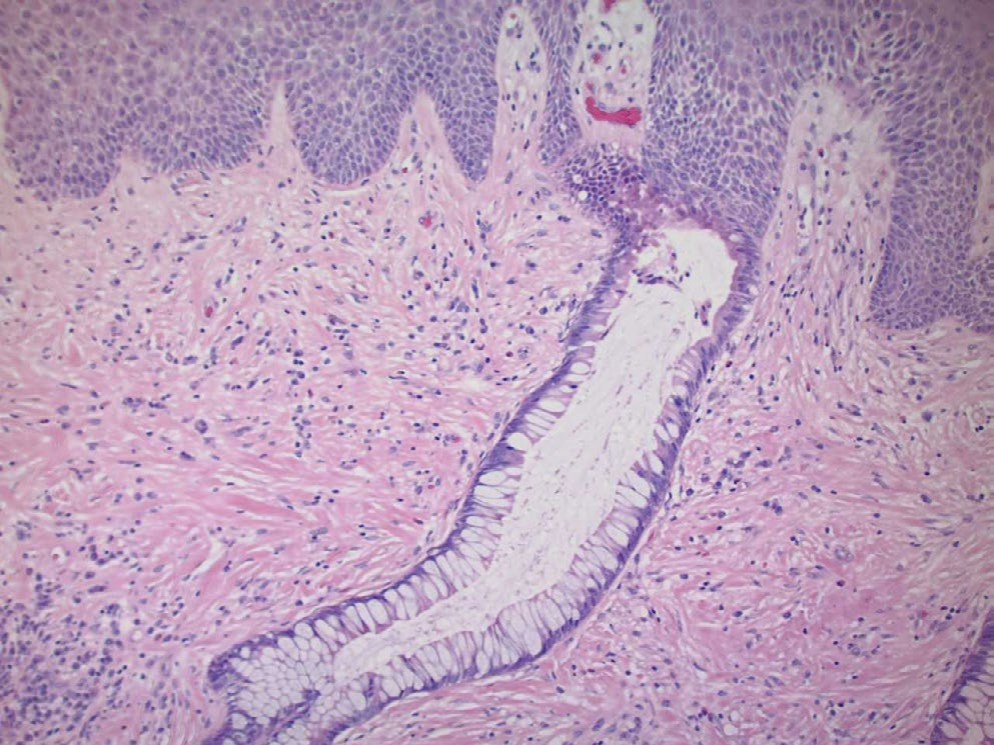
Hematoxylin‐eosin staining of biopsy specimen showing colonic glands merging with the squamous epithelium of the skin (magnification 100×)

A 52‐year‐old man with a history of deep venous thrombosis, iron‐deficiency anemia, gastroesophageal reflux disease well controlled on proton pump inhibitors, and ulcerative colitis (diagnosed at age 13 and is status‐post total proctocolectomy with end ileostomy at age 15) presented to our clinic with a chief complaint of bumps adjacent to his stoma that had been present for less than 6 months. He denied any changes in size or pigmentation. He denied symptoms of pruritus, tenderness, bleeding, or purulent drainage. On review of systems, he denied fevers, chills, oral ulcerations, weight loss, dysphagia, odynophagia, nausea, or vomiting. His ostomy output had been normal, without blood in the effluent in the past 5 years. Patient had no personal history of cancers, but admitted a family history of cutaneous malignancy, specifically basal cell carcinoma in sister, brother, uncle, and father. On his physical examination, his abdomen was soft, flat, nondistended, and nontender, with good bowel sounds in all 4 quadrants. No intraabdominal masses or hepatosplenomegaly were appreciated. However, there were firm mildly hyperkeratotic polypoid nonulcerated papules in the peristomal area of the right lateral abdomen (Figure 1). Differential diagnosis included granulation tissue, verruca vulgaris, and malignancy. A shave biopsy was obtained, and histologic examination revealed squamous epithelium with areas transitioning to enteric columnar mucosa (Figure 2, Figure 3). No evidence of malignant cells was identified on the tissue sections. The pathology finding was deemed consistent with ostomy site‐associated intestinal metaplasia. No further treatment was necessary for the patient since there were no dysplastic features on histology. For management and follow‐up, the results were discussed with the patient's gastroenterology team to educate on the risk of malignant transformation and encourage close surveillance.

## 3 DISCUSSION

There are numerous indications for ostomy creation in complex acute or chronic gastrointestinal disorders, including traumatic injury, inflammatory bowel disease, and malignancy.^6^ Despite diligent stoma care, reports of stomal and peristomal complications such as stenosis, prolapse, retraction, parastomal hernia, parastomal dermatitis, and parastomal bleeding are commonly reported.^6^ There have been few reports of intestinal metaplasia without adenomatous or dysplastic changes adjacent to an ostomy site.^1^, ^2^, ^3^, ^4^ Most of these cases occurred approximately 10 years after ostomy placement, similar to our patient.^1^, ^2^, ^3^, ^4^, ^5^ Predisposing factors that influence cutaneous to intestinal metaplasia remain largely unknown. It has been postulated that chronic inflammation, dynamic change in host bacterial population, or dissemination of intestinal tissue during surgery may play a role.^1^, ^5^

Although rare, some of the reported cases of intestinal metaplasia along ostomy sites have displayed concomitant adenoma or dysplasia in cases of peristomal adenocarcinoma and/or squamous cell carcinoma.^5^, ^7^, ^8^ As such, close clinical monitoring is imperative to identify malignancy early and improve patient morbidity/mortality rates. Clinical presenting symptoms reported in patients with ileostomy carcinomas include palpated mass, small bowel obstruction, peristomal bleeding, pain, stomal prolapse, or retraction.^5^ Evidence of these symptoms should warrant a biopsy or immediate referral to gastroenterology for imaging.

## 4 CONCLUSION

Intestinal metaplasia can occur as a parastomal complication. Fortunately, the risk of malignancy is low, but this possibility necessitates close clinical surveillance. We recommend diligent regular monitoring at a minimum of 10‐15 years postostomy creation as majority of the malignant transformations have occurred within this timeframe. Clinicians should maintain a low threshold for obtaining a biopsy of a parastomal lesion as early identification is imperative in decreasing the risk of malignant conversion.

## CONFLICT OF INTEREST

The authors declare that there are no conflicts of interest regarding the publication of this paper.

## AUTHOR CONTRIBUTIONS

NSU: is a primary case report author and reviewed the literature. MAS: performed histopathology evaluation. MWN: reviewed the case report and is a editor.

## References

[1] Prouty M , Patrawala S , Vogt A , Kelleher M , Lee M , Parker DC . Benign colonic metaplasia at a previous stoma site in a patient without adenomatous polyposis. J Cutan Pathol. 2016;43(3):276‐279. 26453895 10.1111/cup.12638

[2] Patel AN , Kulkarni K , Perkins W . A friable peristomal lesion. Clin Exp Dermatol. 2014;39(3):420‐422. 24635094 10.1111/ced.12287

[3] Ono R , Oka M , Sakaguchi M , et al. Peristomal skin ulcer with intestinal metaplasia. Br J Dermatol. 2012;167(1):204‐206. 22233439 10.1111/j.1365-2133.2012.10819.x

[4] Golubets K , Radu OM , Ho J , Grandinetti LM . Ostomy associated cutaneous colonic metaplasia. J Am Acad Dermatol. 2014;70(1):e18‐e19. 24355281 10.1016/j.jaad.2013.08.051

[5] Quah HM , Samad A , Maw A . Ileostomy carcinomas a review: the latent risk after colectomy for ulcerative colitis and familial adenomatous polyposis. Colorectal Dis. 2005;7(6):538‐544. 16232232 10.1111/j.1463-1318.2005.00807.x

[6] Koc U , Karaman K , Gomceli I , et al. A retrospective analysis of factors affecting early stoma complications. Ostomy Wound Manage. 2017;63(1):28‐32. 28112647

[7] Miida H , Wakaki K , Shimoda S . A rare skin disorder associated with a stoma: intestinal metaplasia and adenoma occurring in a skin ulcer around a colostomy site. Int J Dermatol. 2019;58(9):e173‐e175. 31111465 10.1111/ijd.14506

[8] Johnson CD , White H . Colonic metaplasia with colonic‐type polyps on an ileostomy stoma in polyposis coli. Dis Colon Rectum. 1988;31(5):405‐407. 2835218 10.1007/BF02564900

